# Recent Advances in Chemical Probing Strategies for RNA Structure Determination In Vivo

**DOI:** 10.1002/chem.202503291

**Published:** 2026-01-20

**Authors:** Maryana Yarshova, Jieyu Zhao, Chun Kit Kwok

**Affiliations:** ^1^ Department of Chemistry and State Key Laboratory of Marine Environmental Health City University of Hong Kong Hong Kong SAR China; ^2^ Shenzhen Research Institute of City University of Hong Kong Shenzhen China

**Keywords:** chemical probing, nucleic acids, RNA structure, RNA‐RNA interactions, structure determination

## Abstract

The intricate structures of RNA molecules facilitate their diverse cellular functions. These structures are shaped by the cellular environment, a context that *in silico* and in vitro methods typically cannot reconstitute, making it more difficult to study the structure of RNA in cells. In response to these challenges, RNA structure probing using cell‐permeable chemicals has emerged as an effective method to capture the RNA structural landscape in its native environment. The integration of these probes with advanced adduct detection techniques, particularly second‐ and third‐generation sequencing, has propelled the field forward, facilitating a deeper understanding of the RNA structurome within its precise functional context, including the examination of RNA structure at the single‐molecule and single‐cell levels, within specific subcellular compartments, and across various stages of RNA biogenesis and regulation. This Review summarizes the significant advances in the field of RNA structure probing, focusing on the development of novel structural probes, strategies for RNA structure reconstruction, innovative methodologies that offer extended applicability to address unique biological questions, and concludes with an outlook on future directions in the field.

## Graphical abstract

This Review illustrates how structural probes combined with advanced sequencing technologies are revolutionizing our ability to map RNA structures and interactions within living cells. It highlights the development of innovative strategies tailored to probe RNA structures in particular biological contexts.

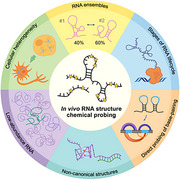

## 1 Introduction

Ribonucleic acid (RNA) has long been a fundamental element of molecular biology, traditionally recognized for its role in the central dogma: messenger RNA (mRNA) conveys genetic information from DNA, ribosomal RNA (rRNA) forms the ribosome's structural and catalytic core, and transfer RNA (tRNA) aids in translating the genetic code into proteins. For years, this protein‐focused perspective dominated, with RNA seen as a mere information carrier or cellular scaffold [1, 2]. However, this view changed as more discoveries followed. This discovery, which was recognized with the 1989 Nobel Prize in Chemistry, provided support for the “RNA World” hypothesis, suggesting that life once existed when RNA served as both genetic material and primary catalytic agent [3]. The realization that RNA could exhibit catalytic activity, shown by self‐splicing introns and the RNA component of RNase P, elevated RNA from passive intermediary to active biochemical participant [4, 5]. In 2000s, the genomic era then revealed that most of the transcriptome in complex organisms does not code for proteins but produces diverse noncoding RNAs (ncRNAs), although their existence was known since 1980s [6, 7, 8], including regulators like microRNAs (miRNAs), long noncoding RNAs (lncRNAs), along with functional elements within mRNAs, including their 5' and 3' untranslated regions (UTRs) [9, 10]. A principle uniting all RNA classes, whether coding or noncoding, is that their biological function depends on their structure, resulting from nucleotide sequence folding. Therefore, elucidating RNA structure is crucial for understanding its functional roles, explaining disease pathogenesis [11], guiding RNA‐targeted therapeutics development [12], enhancing computational predictive models [13], and aiding in evolutionary analysis [14].

Pioneering methods developed for RNA structure elucidation have focused on structural analysis of short, transcript‐specific RNA molecules in vitro. These early studies often focused on abundant transcripts such as tRNA and rRNA, as they are highly expressed in cells and easy to isolate for further analysis [15]. X‐ray crystallography, as one of the first biophysical techniques for RNA structure studies, has proven particularly useful for RNAs that adopt a rigid conformation [16]. Nuclear magnetic resonance (NMR) and, more recently, cryo‐electron microscopy (Cryo‐EM) have demonstrated substantial efficacy as methodologies for the investigation of RNA structural configurations; however, they exhibit a distinct inclination toward the size of RNA molecules, typically favoring smaller entities in the case of NMR, while accommodating larger constructs with Cryo‐EM [17]. Despite the significance progress made, these biophysical techniques are labor intensive, low throughput, and generally applied in vitro [18, 19, 20]. Following biophysical methods, *in silico* methods began to emerge for RNA structure prediction [21, 22]. Although computational methods turned out to be quicker and quite accurate for short‐length RNA molecules, experimental validation is still necessary to confirm the predicted structures, and even more so for longer transcripts, such as those of some mRNAs, lncRNAs or viral genomes [23, 24]. Biochemical methods, such as enzymatic and chemical probing modify RNA bases depending on nucleotide accessibility or reactivity to these probes, which indicates their propensity to be in either paired or unpaired state offer an alternative method for RNA structure determination [25]. Advantages of enzymatic probing include their structural and/or sequence specificity and application in biological buffers closely resembling physiological conditions [26]. However, large size of enzymatic probes contributes to potential information loss due to steric hindrance, and limits their use to in vitro conditions only as they are unable to cross the cell membrane [26, 27].

**FIGURE 1:**
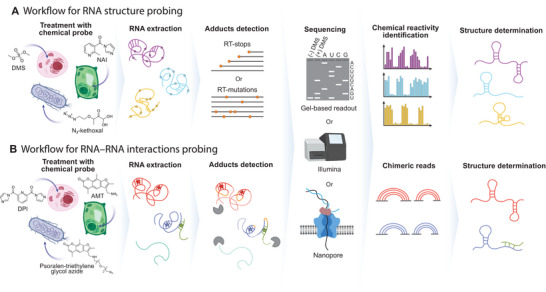
General workflow overview of in vivo structure probing of the RNA. (A) Accessible nucleotides are covalently modified using structure probes, followed by RNA extraction from cells. Covalent adducts on RNA are then detected through RT‐stops or RT‐mutations, followed by sequencing method of choice. The position of the covalent adduct on the RNA sequence allows identification of reactivity at single‐nucleotide resolution, which can then be used as an experimental constraint to guide RNA structure determination. Illustrated structure probes include dimethyl sulphate (DMS), 2‐methylnicotinic acid imidazole (NAI), and azide‐kethoxal (N_3_‐kethoxal). (B) Probes for RNA‐RNA interaction detection were applied to live cells with subsequent RNA extraction. Adducts on cross‐linked RNA can be identified with a method of choice, such as RNase treatment and proximity ligation, producing chimeric fragments that are read out by sequencing. Structure probes used as example: 4’‐aminome‐thyltrioxaie (AMT), amotosalen, dipicolinic acid imidazolide (DPI).

Over the past decades, numerous studies highlighted the differences between in vitro and in vivo RNA structures [28, 29]. Such differences are attributed to the failure to reconstitute the precise cellular environment that alters RNA structure under in vitro condition, including: the presence of RNA‐binding proteins, small‐molecule metabolites, differences in ionic conditions, and cellular crowding. Since their initial applications, chemicals and chemical probes, among the earliest RNA structural probes developed, consistently gained attention due to their compact size, several RNA modification mechanisms, and potential for easier cell permeability [15]. These structural probes can effectively target distinct nitrogenous bases (i.e. base‐specific) or act in a nonbase specific manner [15]. In recent years, the increasing demand for high‐throughput and improved resolution of RNA structure determination has led to the integration of chemical probing with second‐ or third‐generation sequencing, shifting the focus from gel electrophoresis (Figure 1). This Review highlights significant advances in the structure probing of RNA and RNA‐RNA interactions in vivo using small molecules. We begin by examining the chemistries of the developed probes and approaches for the analysis of chemical probing data, followed by a collection of methods developed specifically for in vivo RNA structure probing, first at the transcript‐specific level and then at the transcriptome‐wide level, with a focus on techniques leveraging second‐ and third‐generation sequencing. Within these sections, we also detail innovative approaches designed to investigate biological questions aimed at investigating specific aspects of the RNA structurome, such as structural analysis of targeted RNAs, specific steps in RNA regulation, and different levels of organizational complexity. Given the concise nature of this review, RNA‐protein interactions will not be discussed and can be found in other excellent reviews [30, 31, 32, 33].

## 2 Chemicals and Chemical Probes

### 2.1 Base‐specific Probes

**FIGURE 2:**
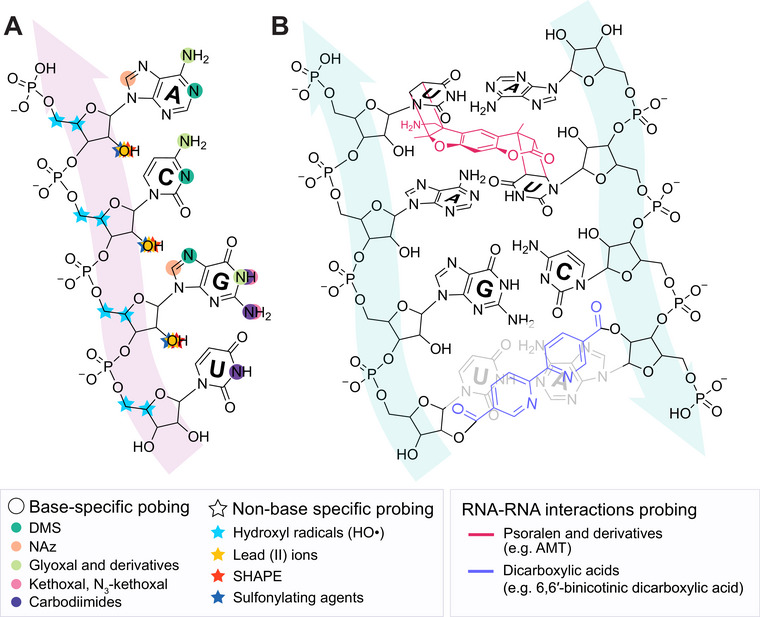
Sites of adduct formation for base‐specific probes and nonbase specific chemicals and chemical probesused to interrogate RNA structure (A) and RNA‐RNA interactions (B) in vivo. (A) Base‐specific probes (e.g., dimethyl sulphate (DMS; dark green), nicotinoyl azide (NAz; peach), glyoxal, kethoxal and their derivatives (light green and pink), carbodiimides (e.g. ETC, EDC; purple)) covalently modify particular nitrogenous bases. (B) Nonbase specific chemicals and chemical probes (e.g., hydroxyl radicals (HO•; light blue), lead (II) ions (Pb^2+^; yellow), SHAPE (e.g. NAI, 2A3, etc.; red) and sulfonylating agents (e.g. PS3; dark blue)) modify or cleave the sugar‐phosphate backbone. Psoralen (e.g. AMT; mangenta) and dicarboxylic acids (e.g. 6,6’‐binicotinic dicarboxylic acid; light purple) are shown as examples of probes for RNA‐RNA interactions probing.

Dimethyl sulphate (DMS) was the first base‐specific probe applied in vivo in *Escherichia coli* cells, methylating N1 of adenines and N3 of cytosines on Watson and Crick face of unpaired residues (Table 1, Figure 2) [34, 35]. For guanines, DMS preferentially methylates N7 position on Hoogsteen face, making it more suitable for analysis of noncanonical RNA structures, such as G‐quadruplex (G4), rather than determining canonical RNA base‐pairing interactions [36]. It was later demonstrated that mildly alkaline conditions could facilitate probing of base‐pairing face of uracil and guanine in cells and in vitro respectively [37]. Probing of Watson and Crick face of guanine by DMS in living cells initially posed a challenge due to the preference of DMS for N7 methylation, which occurred irrespective of the base‐pairing state. Nevertheless, it was rendered feasible through the use of mutational profiling (MaP), particularly a distinctive mutational signature of N1‐methylguanine, enabling four‐base DMS probing [38]. Despite DMS being favored for its cell permeability, it is a highly toxic chemical which can lead to RNA degradation, therefore careful handling and optimization of DMS concentration and treatment time is necessary for in vivo tests [39, 40]. Nevertheless, it remains one of the most commonly employed RNA structure‐probing reagents because of its versatility for both in vitro and in vivo applications, rapid cell penetration, single‐nucleotide resolution, compatibility with downstream analysis, integration into computational modeling tools, and commercial availability.

**TABLE 1:** Structural probes used for in vivo RNA structure probing. Comprehensive overview of structural probes, categorized as base‐specific, nonbase specific, and RNA‐RNA interaction probes. For each reagent, key information is presented, including the CAS number and commercial availability, chemical structure, adduct formation site, remarks on its use and properties, and key references.

Base‐specific probes	CAS Number, commercial availability^[a]^	Chemical structure	Specificity	Remarks	Key references
Dimethyl sulphate (DMS)	77‐78‐1, Yes	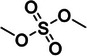	Watson‐Crick face of N1 of adenines and N3 of cytosines not involved in Watson‐Crick interactions, Hoogsteen face of N7 of guanines	‐ DMS does not typically react with the Watson‐Crick face of guanine and uridine nucleotides due to protonation of the respective N1 and N3 positions at neutral pH. ‐ Can be used to probe all four bases at pH = 8, promoting transient deprotonation of guanine and uridine ‐ Does not require a permeabilization agent to cross cell membranes	[37, 107]
Nicotinoyl azide (NAz)	4013‐72‐3, Yes	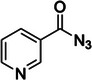	C8 of solvent‐accessible guanines and adenines	‐ Used in LASER (Light‐Activated Structural Examination of RNA).	[49]
6‐(azidomethyl)nicotinoyl azide (NAz‐N_3_)	N/A, N/A	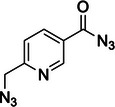	C8 of solvent‐accessible guanines and adenines	‐ Used in icLASER (in vivo click Light‐Activated Structural Examination of RNA).	[125]
Glyoxal and glyoxal derivatives					
Glyoxal	107‐22‐2, Yes	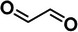	Watson‐Crick face of unpaired guanines >> cytosines and adenines	‐ Reveals both RNA base‐pairing and protein footprinting in vivo. ‐ Requires Gs to populate the rare anionic state.	[43]
Methylglyoxal	78‐98‐8, Yes	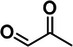	Watson‐Crick face of unpaired guanines >> cytosines and adenines	‐ Higher specificity for guanines over cytosines compared to glyoxal. ‐ Extend of modification of guanines is higher than for glyoxal. ‐ Higher hydrophobicity may enhance membrane transversing.	[43]
Phenylglyoxal	1074‐12‐0, Yes	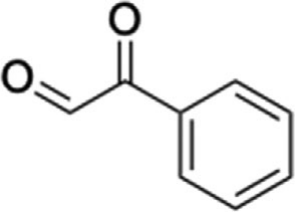	Watson‐Crick face of unpaired guanines >> cytosines and adenines	‐ Higher specificity for guanines over cytosines compared to glyoxal. ‐ Extend of modification of guanines is higher than for glyoxal and methyl glyoxal. ‐ Higher hydrophobicity may enhance membrane transversing. ‐ Larger bulk of phenylglyoxal may enable it to better position a carbonyl near N1 of guanine relative to methylglyoxal	[43]
3‐(2‐Azidoethoxy)‐1,1‐dihydroxy‐2‐butanone (N_3_‐kethoxal)	2382756‐48‐9, Yes	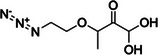	Watson‐Crick face of N1 and N2 of unpaired guanines	‐ Lack of synthetic routes to modified kethoxal hampered its use for transcriptome‐wide studies. ‐ New synthetic route was identified to incorporate azide bioorthogonal handle that can be enriched further.	[44]
Carbodiimides					
1‐ethyl‐3‐(3‐dimethylaminopropyl)carbodiimide (EDC)	N/A, N/A	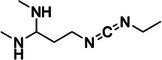	Watson‐Crick face of unpaired N3 of uracil > N1 and N2 of guanine	‐ Uracil is preferred over guanine with a factor of ≈1.5 ‐ Unlike glyoxal, EDC can react with guanines in their neutral state ‐ Useful in small concentrations for RT stops experiments, but can lead to RNA degradation at high concentration required for MaP	[47, 134]
1‐ethyl‐3‐(3‐dimethylaminopropyl) carbodiimide methiodide (ETC)	22572‐40‐3, Yes	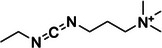	Watson‐Crick face of N1 and N2 of unpaired guanines and N3 of uracils	‐ Developed to overcome RNA damaging issue produced by EDC at high concentrations required for MaP.	[48]
					
**Nonbase specific chemicals**					
Hydroxyl radical	3352‐57‐6, Yes	HO•	C4 and C5 of ribose	‐ Requires the use of synchrotron radiation ‐ Reacts with hydrogens on C4 and C5 of ribose that are accessible to solvent to induce cleavage.	[135, 136]
Lead (II) ion	14280‐50‐3, Yes	Pb^2+^	2'‐OH of ribose	‐ Probing by cleavage of phosphodiester bond ‐ Cleavage can occur either at or near the metal binding‐site (rarely observed) or at single‐stranded RNA regions (more frequently observed).	[109, 136]
					
SHAPE					
2‐methylnicotinic acid imidazolide (NAI)	1055970‐47‐2, Yes	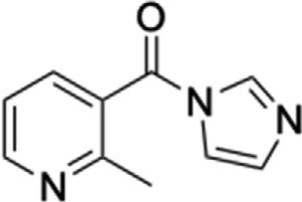	2'‐OH of ribose	‐ Electron‐deficient character of the pyridine ring increases the electrophilicity of the carbonyl group.	[56]
2‐methylnicotinic acid imidazolide azide (NAI‐N_3_)	1612756‐29‐2, Yes	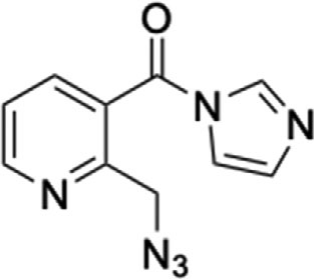	2'‐OH of ribose	‐ Functionalized with azido moiety which allows for click‐chemistry application in vivo.	[60]
2‐methyl‐3‐furoic acid imidazolide (FAI)	1415238‐77‐5, Yes	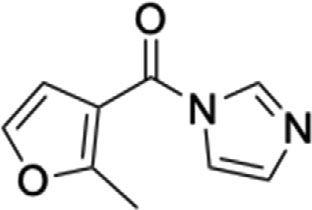	2'‐OH of ribose	‐ Less reactive than NAI.	[56]
2‐methyl‐3‐furoic acid imidazolide azide (FAI‐N_3_)	N/A, N/A	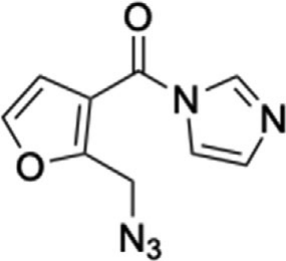	2'‐OH of ribose	‐ Developed as analogous probe to NAI‐N_3_ as nicotinoyl scaffold is highly prone to hydrolysis.	[61]
1‐methyl‐7‐nitroisatoic anhydride (1M7)	73043‐80‐8, Yes	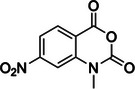	2'‐OH of ribose	‐ Developed as a fast‐acting SHAPE reagent to increase reaction time compared to NMIA. ‐ Also more labile toward advantageous, self‐limiting, hydrolysis.	[58, 137]
2‐aminopyridine‐3‐carboxylic acid imidazolide (2A3)	2765091‐45‐8, Yes	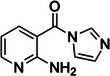	2'‐OH of ribose	‐ Outperforms NAI when probing RNA in vivo.	[62]
Acetylimidazole (AcIm)	2466‐76‐4, Yes	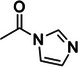	2'‐OH of ribose	‐ Provide enhanced signals for base modifications inaccessible to NAI‐N_3_ due to its larger size.	[63]
Acetylimidazole azide (AcIN_3_)	N/A, N/A	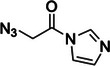	2'‐OH of ribose		[63]
5‐nitroisatoic anhydride (5NIA)	4693‐02‐1, Yes	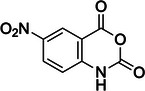	2'‐OH of ribose	‐ Showed higher reactivity rates with adenosine yet discriminates well between paired and unpaired regions for all nucleotides.	[57, 94]
1‐methyl‐6‐nitroisatoic anhydride (1M6)	4693‐01‐0, Yes	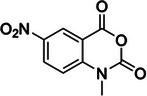	2'‐OH of ribose	‐ While was applied in vivo, it is usually preferred for in vitro use.	[138]
N‐methylisatoic anhydride (NMIA)	10328‐92‐4, Yes	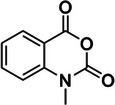	2'‐OH of ribose	‐ While was applied in vivo, it is usually preferred for in vitro use.	[56]
3‐((1H‐1,2,4‐triazol‐1‐yl)sulfonyl)pyridine (P3S)	2988852‐68‐0, Yes	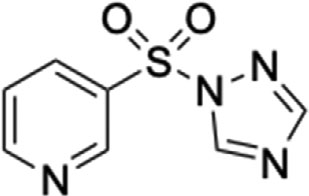	2'‐OH of ribose	‐ Sulfonylation works analogous to SHAPE chemistry, yet reagents with sulfonyl moiety have enhanced stability in aqueous solution.	[64]
					
**Probes for RNA‐RNA interactions**					
Psoralen‐derivatives cross‐linkers					
4'‐aminomethyl‐4,5',8‐trimethylpsoralen hydrochloride (AMT)	62442‐61‐9, Yes	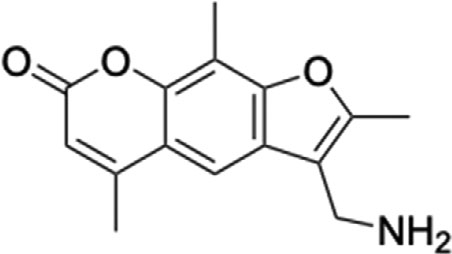	Preference for staggered uridines	‐ One of the first psoralen derivatives designed.	[67, 139]
Amotosalen (also known as S‐59)	161262‐29‐9, Yes	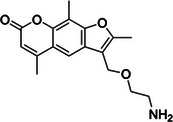	Preference for staggered uridines	‐ More soluble than AMT.	[68]
Psoralen‐triethylene glycol azide	1352815‐11‐2, Yes	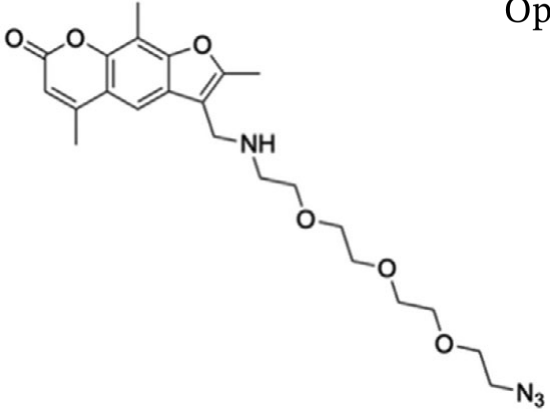	Opposing uridine bases	‐ Designed for the use in COMRADES protocol	[69]
Bifunctional acylators					
Dipicolinic acid imidazolide (DPI)	N/A, N/A	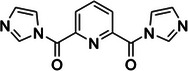	2'‐OH of ribose	‐ Faste hydrolysis rate than NAI is observed due to the presence of additional electron‐withdrawing group. ‐ Suitable for rapid RNA cross‐linking.	[70]

^a^ “Commercial availability” refers to catalog availability for standard chemical supply companies as of October 2025.

Notably, the development of probes like glyoxal and kethoxal for in vivo RNA profiling has been a more recent endeavor. The in vivo application of glyoxal was conducted alongside its derivatives: methylglyoxal and phenyl glyoxal, with phenylglyoxal exhibiting the highest extent of reactivity with guanines, but also reacting with adenines and cytosines [41, 42, 43]. As a hydrophilic molecule, kethoxal was only applied in vivo in lysolecithin‐permealized cells, with the lack of synthetic modification routes hindering its further applications [44, 45]. The subsequent development of the azido‐modified kethoxal (N_3_‐kethoxal) installed bioorthogonal azide handle enabling click‐chemistry for downstream analysis and solved the limitation of cell permeability, as it was shown to efficiently enter living cells for transcriptome‐wide RNA structure probing [44, 46].

Carbodiimides, characterized by the functional group RN = C = NR, have proven to be versatile RNA structure probing reagents due to their selectivity toward flexible uracils and guanines (Table 1, Figure 2) [47]. The first carbodiimide applied in vitro was 1‐cyclohexyl‐3‐(2‐morpholinoethyl)carbodiimide metho‐p‐toluenesulfonate (CMCT), however, its poor membrane permeability rendered it inapplicable in live cell contexts [45]. Two new chemical reagents, 1‐ethyl‐3‐(3‐dimethylaminopropyl)carbodiimide (EDC) and 1‐ethyl‐3‐(3‐dimethylaminopropyl) carbodiimide methiodide (ETC), were designed to overcome this limitation and were successfully applied in vivo [47, 48]. It is noteworthy that higher concentrations of EDC can cause RNA degradation, limiting its adduct detections to RT stops only [48].

Lastly, nicotinoyl azide (NAz) represents a class of light‐activated base‐specific probes implemented in the light‐activated structural examination of RNA (LASER), the specific advantage of which lies in its ability to control its activation using UV light. NAz modifies C8 of solvent‐accessible guanines and adenines both in vitro and in living cells (Table 1, Figure 2); however, unlike other chemical probes, it is not limited to single‐stranded regions only, forming adducts on both single‐ and double‐stranded regions. It therefore makes it a useful and unique complement to other structural probes [49].

### 2.2 Nonbase‐Specific Chemicals

The first two nonbase‐specific chemicals, hydroxyl radicals (HO•) and lead (II) ions (Pb^2+^), were introduced in vitro, yet since then were adapted for in vivo use [50, 51, 52, 53]. Hydroxyl radicals cleave the RNA backbone at nucleotide resolution by abstracting hydrogen atoms from the sugar phosphate, a process that is strictly dependent on solvent accessibility [54]. It is usually referred to as the gold standard for probing RNA structure based on solvent accessibility, yet its implementation into in vivo studies remains challenging due to synchrotron radiation necessary for radicals generation [54]. Lead (II) ions, in the form of lead (II) acetate, readily penetrate cells and induce partial cleavage of RNA at solvent‐accessible loops, bulges, and single‐stranded regions [53]. While hydroxyl radicals are valuable for investigating the 3D folding of RNA structures, lead (II) ions provide insights into RNA secondary structures, making them useful both individually and as complementary tools (Table 1, Figure 2).

Another category of nonbase‐specific probes are SHAPE (Selective 2′‐Hydroxyl Acylation Analyzed by Primer Extension) reagents, which are small molecule electrophiles that selectively acylate the 2′‐OH group of flexible, unconstrained ribose sugars in RNA, thereby providing insights into nucleotide flexibility and secondary RNA structure [55]. To date, more than ten different SHAPE reagents (Table 1) have been identified as suitable for in vivo use, each possessing unique physiochemical properties, including molecular size, presence or absence of an azide handle, reaction mechanism, and hydrolysis half‐life (Figure 2). Early SHAPE reagents, such as N‐methylisatoic anhydride (NMIA), are more suited to in vitro studies due to low aqueous solubility and cross‐reactivity with other nucleophiles in living cells [56]. Its successor, 1‐methyl‐7‐nitroisatoic anhydride (1M7), while also having relatively short reaction half‐life, has proven useful for in vivo structure probing due to its lack of bias for particular nucleotides and ease of synthesis [57, 58, 59]. Importantly, these two probes were initially designed for in vitro SHAPE probing, which then began to emerge as an RNA structure probing approach.

The goal to develop soluble and 2’‐OH specific SHAPE reagents for RNA structure probing suitable for living cells has yielded a range of acyl imidazole probes (Table 1, Figure 2). First two of such molecules, 2‐methylnicotinic acid imidazolide (NAI) and 2‐methyl‐3‐furoic acid imidazolide (FAI), were intentionally designed to have increased solubility thanks to the implementation of heteroatoms in aromatic rings, as well as low‐toxicity imidazole as leaving group for tuning reactivity without affecting solubility [56]. Hydrolysis half‐lives of NAI and FAI resulted to be 33 min and 73 min respectfully, which is considerably longer than 8 min half‐life of NMIA, allowing for enhanced acylation efficiency of RNA in vivo owing to longer possible reaction time [56]. Subsequent development of dual‐functioning azide‐functionalized NAI and FAI probes, termed NAI‐N_3_ and FAI‐N_3_, enabled selective RNA enrichment via bioorthogonal click chemistry subsequently reducing background of RNA structure probing experiments [60, 61]. Further studies focused on developing SHAPE probes with enhanced performance, one of such being 2‐aminopyridine‐3‐carboxylic acid imidazolide (2A3). Its optimized steric and electronic properties increase membrane permeability and yield a higher signal‐to‐noise ratio and more accurate RNA structural predictions than NAI in vivo, making it a promising SHAPE probe [62]. Most recent development includes acetyl imidazole (AcIm) and acetyl imidazole azide (AcIN_3_), small size of which permits access to folded RNA structure and therefore provide a broader coverage of intracellular RNA structure, previously inaccessible to other probes [63].

Additionally, recently developed sulfonylation agents have been designed as alternatives to SHAPE probing, achieving single‐nucleotide resolution both in vitro and in vivo (Table 1, Figure 2) [64]. This new reagent class fundamentally expands the chemistry of RNA 2'‐OH modification beyond activated carbonyls to include sulfonyl electrophiles, forming stable O‐sulfonate ester adduct, capable of inducing RT‐stops. A key advantage of these small‐molecules is their high aqueous solubility and stability, demonstrating longer reaction half‐lives in aqueous buffer reaching up to 7 days for some compounds as compared to 34 min of NAI [64]. Similar to SHAPE reagents, sulfonylating agents are cell‐permeable and preferentially modify unpaired RNA regions over helices. However, the current generation of sulfonylating agents exhibits lower intrinsic reactivity compared to acyl imidazoles, requiring longer incubation times (e.g. 1 h for PS3, as compared to 5 min for NAI) for sufficient signal, which could potentially perturb cellular processes during probing [64]. Furthermore, the reactivity profile of sulfonylating agents is not identical to that of SHAPE reagents, potentially because of the differences in the tetrahedral geometry of the sulfonyl centre and the electronic properties that yield distinct modification patterns on the same RNA, suggesting that sulfonylation and acylation could provide complementary structural insights rather than serve as a complete substitute for SHAPE probing.

### 2.3 Probes for RNA‐RNA Interactions

Psoralen and psoralen derivatives serve as cross‐linkers in the investigation of RNA‐RNA interactions, covalently binding to opposing uracil bases within either a single RNA molecule or between two separate RNA molecules upon irradiation with UV light [65]. The first recorded use of psoralen cross‐linking in vitro was in 1977, followed by in vivo studies in 1979, which employed the reversible psoralen derivative 4′‐aminomethyl‐4,5′,8‐trimethylpsoralen (AMT) in HeLa cells (Table 1, Figure 2) [66, 67]. AMT has since been used extensively for RNA‐RNA interactions probing in a wide range of studies, some of which are also discussed below. The more recent development of amotosalen (S59) represents a further refined psoralen derivative, offering improved water solubility and therefore higher cross‐linking efficiency [68]. Aiming to increase functionality of psoralen probes, azide modification was installed on AMT, allowing for biotin ligation and pull‐down for further enrichment, without affecting cross‐linking efficiency [69].

A distinct category of RNA‐RNA interaction probes includes a subtype of SHAPE reagents known as bifunctional acylators. These molecules, equipped with flexible linkers, facilitate the cross‐linking of spatially proximal RNA nucleotides by targeting the 2’‐OH group on ribose. In the seminal study conducted by Ryan Van Damme et al., eight bifunctional reversible acylators were synthesized in one‐step reaction [70]. Notably, three of these compounds, terephthalic, isocinchomeronic, and dipicolinic acid imidazolide (DPI) (Table 1, Figure 2), achieved cross‐linking efficiencies of 97–99%. Furthermore, the measured hydrolysis lifetime of DPI indicated a faster reaction time than that of the commonly used in vivo SHAPE reagent 2‐methylnicotinic acid imidazolide (NAI), suggesting rapid RNA cross‐linking capability.

## 3 Data Interpretation and Methods for In Vivo RNA Structure Probing

### 3.1 RNA Structure Reconstruction

Data collected from RNA structure probing experiments is then used decipher RNA structure. Transcript‐specific RNA structure probing primarily relies on identifying reverse transcription (RT) signatures, which can be categorized into RT stops (premature termination of cDNA synthesis) or RT mutations (mismatches incorporated during cDNA synthesis) depending on different characteristic of reverse transcriptases [71, 72]. Modification sites from structure probing can then be determined using gel‐based readout and, second‐or third‐generation sequencing (Table 2). Resolving cDNA fragments after reverse transcriptase treatment on sequencing gels and quantifying bands is a traditional approach and still widely used today for structure identification for short transcripts or transcript regions. Reverse transcriptase stalls directly trace to modification sites produced by RNA structure probes and provide a measure of the nucleotide position being paired or unpaired (i.e. low and high raw reactivity respectively). For approaches relying on RT mutations, mutational profile from DNA sequencing or direct detection of modifications with third‐generation sequencing platforms can similarly be converted to reactivity profiles and implemented into *in silico* RNA structure determination. Obtained raw reactivity profiles can be normalized and used as constrains after conversion to pseudo‐free energy change terms for thermodynamics‐dependent or thermodynamics‐independent computational approaches, significantly improving accuracy of computational RNA structure [73, 77]. Examples of softwares suitable secondary RNA structure using implementation of experimental RNA structure probing data include RNAstructure, SeqFold, ShapeKnots, Vienna RNA websuite, SimRNAweb v2.0, while ShapeMapper2 acts as an example of software used for determining per‐nucleotide reactivity profiles [78, 79, 80, 81, 82, 83].

**TABLE 2:** Methods for the determination of in vivo RNA structure. Overview of transcript‐specific and transcriptome‐wide methods for probing RNA structure in vivo compared by chemistry, year of introduction, detection strategy, sequencing platform, experimental conditions, and organisms used for in vivo studies.

Method	Chemistry	Year	Modifications detection	Sequencing	Conditions tested in	Organism(s) tested in^a^	Key references
**Transcript‐specific**							
Lead(II) cleavage	Lead(II) backbone cleavage	2002	RT‐stops	Gel‐based readout	in vivo	E. coli	[53]
Hydroxyl‐radical footprinting	•OH backbone scission	2006	Ligation‐mediated 5ʹ mapping	Gel‐based readout	in vitro in vivo	E. coli	[136]
DMS/SHAPE‐LMPCR	DMS or SHAPE	2013	RT‐stops	Gel‐based readout	in vitro in vivo	A. thaliana	[92]
Mod‐Seq	Any small‐molecule probe	2014	RT‐stops	Illumina	in vivo	S. cerevisiae	[95]
Targeted Structure‐Seq	DMS	2015	RT‐stops	Illumina	in vivo	M. musculus	[96]
In‐cell SHAPE‐Seq	SHAPE	2016	RT‐stops	Illumina	in vitro in vivo	E. coli	[93, 140]
LASER	NAz	2017	RT‐stops	Gel‐based readout	in vitro in vivo	H. sapiens	[49]
LASER‐Seq/MaP	NAz	2018	RT‐stops/RT‐mutations	Illumina	in vitro in vivo	H. sapiens	[102]
PAIR‐MaP	DMS	2019	RT‐mutations	Illumina	in vitro in vivo	H. sapiens E. coli	[37]
Sulfonylation probing	2ʹ‐OH sulfonyl fluoride	2022	RT‐stops	Gel‐based readout	in vitro in vivo	*H. sapiens*	[64]
smStructure‐seq	SHAPE	2022	RT‐mutations	PacBio	in vivo	*A. thaliana*	[87]
Nano‐DMS‐MaP	DMS	2023	RT‐mutations	Nanopore	in vitro in vivo	*H. sapiens*	[101]
DANCE‐MaP	DMS	2023	RT‐mutations	Illumina	in vitro in vivo	*H. sapiens*	[100]
mitoDMS‐MaPseq	DMS	2024	RT‐mutations	Illumina	in vivo	*H. sapiens*	[119]
							
**Transcriptome‐wide**							
DMS‐Seq	DMS	2013	RT‐stops	Illumina	in vitro in vivo	*S. cerevisiae*	[103]
Structure‐Seq	DMS	2013	RT‐stops	Illumina	in vivo	*A. thaliana*	[104]
icSHAPE	NAI‐N_3_	2015	‐stops	Illumina	in vitro in vivo	*M. musculus*	[60]
DMS‐MaPSeq	DMS	2016	RT‐mutations	Illumina	in vivo	*S. cerevisiae* *H. sapiens*	[105]
PARIS	AMT‐psoralen	2016	Enzymatic fragmentation	Illumina	in vivo	H. sapiens *M. musculus*	[141]
SPLASH	Psoralen	2016	Enzymatic fragmentation	Illumina	in vivo	*E. coli* *H. sapiens* S. cerevisiae	[120]
LIGR‐Seq	Psoralen	2016	Enzymatic fragmentation	Illumina	in vivo	*H. sapiens*	[121]
SHALiPE‐Seq	SHAPE	2016	Lithium ion (Li^+^)‐based primer extension (LiPE)	Illumina	in vitro in vivo	*A. thaliana*	[111, 142]
Structure‐Seq2	DMS	2017	RT‐stops	Illumina	in vivo	*O. sativa*	[106]
SPET‐Seq	DMS	2017	RT‐stops	Illumina	in vitro in vivo	*E. coli*	[113]
SHAPE‐MaP	SHAPE	2018	RT‐mutations	Illumina	in vivo	*E. coli* *H. sapiens* *M. musculus*	[94, 143]
COMRADES	Psoralen	2018	Enzymatic fragmentation	Illumina	in vivo	*H. sapiens*	[69]
Keth‐Seq	N_3_‐Kethoxal	2019	RT‐stops	Illumina	in vitro in vivo	*M. musculus* *H. sapiens*	[44]
Cap‐Structure‐Seq	NAI	2020	RT‐stops	Illumina	in vivo	*A. thaliana*	[115]
Lead‐Seq	Lead(II)	2020	Ligation‐mediated 5ʹ end capture	Illumina	in vivo	*Y. pseudotuberculosis*	[109]
icSHAPE‐MaP	NAI‐N_3_	2021	RT‐mutations	Illumina	in vitro in vivo	*H. sapiens*	[123]
PARIS2	Amotosalen	2021	Enzymatic fragmentation	Illumina	in vivo	*H. sapiens* *M. musculus*	[68]
PORE‐cupine	NAI‐N_3_	2021	Nanopore	Nanopore	in vitro in vivo	*H. sapiens* *B. subtilis*	[98]
icLASER	NAz‐N_3_	2021	RT‐stops	Illumina	in vitro in vivo	*H. sapiens*	[125]
SHARC	DPI	2022	Reversible crosslinking	Illumina	in vitro in vivo	*H. sapiens*	[70]
smartSHAPE	SHAPE	2022	RT‐stops	Illumina	in vivo	*M. musculus*	[124]
sc‐SPORT	DMS	2023	RT‐mutations	Illumina	in vitro in vivo	*H. sapiens*	[117]
tRNA‐Structure‐Seq	DMS	2023	RT‐stops	Illumina	in vitro in vivo	*E. coli*	[116]
KARR‐seq	N_3_‐Kethoxal	2024	Pull‐down and proximity ligation	Illumina	in vitro in vivo	*H. sapiens* *M. musculus* *D. melanogaster*	[46]
DMS‐FIRST‐seq	DMS	2025	Nanopore	Nanopore	in vitro in vivo	*H. sapiens* *E. coli*	[144]
DM‐DMS‐MaPseq	DMS	2025	RT‐mutations	Illumina	in vitro in vivo	*H. sapiens*	[118]
CoSTseq	DMS	2025	RT‐mutations	Illumina	in vivo	*Y. pseudotuberculosis*	[114]

Abbreviations: DMS – dimethyl sulphate, SHAPE – Selective 2'‐hydroxyl acylation analysed by primer extension; NAI – 2‐methylnicotinic acid imidazolide; NAz – nicotinoyl azide; RT‐mutations – reverse transcriptase mutations;RT‐stops – reverse transcriptase stops.

^a^ Abbreviations for organisms tested in: Arabidopsis thaliana (A. thaliana), Bacillus subtilis (B. subtilis), Drosophila melanogaster (D. melanogaster), Escherichia coli (E. coli), Homo sapiens (H. sapiens), Mus musculus (M. musculus), Oryza sativa (O. sativa), Saccharomyces cerevisiae (S. cerevisiae), Yersinia pseudotuberculosis (Y. pseudotuberculosis).

Importantly, the rise of high‐throughput sequencing allowed for reads to be clustered, aiding in structure reconstruction of all of the RNA structural states present in solution rather than the average structure, i.e. RNA ensemble deconvolution. In such cases, the number of identified clusters is directly related to the number of RNA structures. Beyond defining intramolecular folds, the reactivity profiles from structure probing are also powerful constraints for predicting RNA‐RNA interactions [84]. The propensity of a nucleotide to be unpaired, quantified as its unpaired probability, defines its potential to interact with another RNA molecule. Computational tools like IntaRNA can incorporate SHAPE or DMS reactivity data by converting it into pseudo‐energy terms that guide the identification of accessible interaction sites [85, 86]. Most notable algorithms include RNA interaction groups by mutational profiling (RING‐MaP), Determination of the Variation of the RNA structure conformation through stochastic context‐free grammar (DaVinci), Deconvolution of RNA Alternative Conformations (DRACO), Detection of RNA folding ensembles using expectation‐maximization (DREEM), and Deconvolution and Annotation of riboNucleic Conformational Ensembles measured by Mutational Profiling (DANCE‐MaP) [87, 88, 89, 90, 91].

### 3.2 Transcript‐specific Methods

A collection of structural probes discussed in the section above was successfully integrated into protocols that detect the modifications or cleavage sites created by these probes. The techniques described below refine specific aspects of the RNA structure probing workflow, driven by the need to overcome specific limitations, such as low abundance, structural heterogeneity, or the necessity to link structure to function in a complex cellular milieus. As such, these techniques become tailored to solve RNA structure within particular biological context.

#### 3.2.1 Probing Low Abundance and Targeted Transcripts

The study of low‐abundance RNAs is complicated by their scarcity, which masks their structural signals in bulk assays. To overcome this challenge, DMS/SHAPE‐LMPCR was developed and tested in *Arabidopsis thaliana* by combining DMS or SHAPE chemistry with ligation‐mediated PCR (Table 2). A key advantage of the method was its 100,000‐fold increase in sensitivity over other RT‐based methods, thereby unlocking the structural landscape of low‐abundance transcripts [92]. Concurrently, other targeted strategies have been established for bacterial and mammalian systems. In‐cell SHAPE‐seq was developed to simultaneously characterize RNA structure and function in bacterial cells, by combining SHAPE probing with a specialized two‐plasmid system allowing to observe changes in gene expression for two translation regulating mechanisms [93]. By providing a different redout method, in‐cell SHAPE MaP further increased the sensitivity and accuracy of the approach by avoiding multiple ligation step and, simplifying the chemical probing readout. Adding to the in‐cell SHAPE‐seq, this method is suitable for probing RNA structure in vivo in both bacterial and mammalian cells [72, 94]. Mod‐seq, which allows for the use of any chemical probe causing RT‐stops, further expanded the field's capability to profile diverse RNAs, as illustrated by structure probing of four ribosomal and thirty two noncoding yeast RNA [95]. Similarly, Targeted Structure‐Seq was created to probe the conformation of specific, large noncoding RNAs directly in cells by combining in vivo DMS probing with target‐specific reverse transcription and deep sequencing, enabling high‐resolution structural analysis of full‐length, low‐abundance RNAs like the Xist long noncoding RNA (lncRNA) [96]. Taken together, these methods provide a way to study the structure of RNA transcripts of interest and, potentially, the downstream structure‐function relationship in RNA biology. A persisting challenge, however, is that short‐read sequencing struggles to assign structural data to specific transcript isoforms or capture the connectivity between distant structural domains.

Methods such as first strand sequencing (DMS‐FIRST‐Seq) and RNA structure analysis using nanopore sequencing (PORE‐cupine) directly address this limitation by utilizing nanopore sequencing (Table 2). DMS‐FIRST‐Seq leverages long reads to deconvolve entire structural ensembles from a population of RNAs, identifying and quantifying coexisting alternative conformations for a single transcript sequence that would be averaged into a single signal with short reads [97]. PORE‐cupine detects chemical modifications directly from the nanopore current signal, bypassing rever​se transcription and PCR entirely. This allows it to phase structural information along full‐length transcripts, revealing how the same sequence can fold differently in alternative isoforms of the same gene, with direct functional consequences on processes like translation [98]. Collectively, the strategic integration of long‐read sequencing platforms is pushing the field toward a more nuanced understanding of the RNA structurome, enabling the capture of full‐length, isoform‐specific structural information within a native cellular context.

#### 3.2.2 Deconvoluting Structural Heterogeneity and Dynamics

The notion of RNA existing as a diverse ensemble of conformations is often overlooked by traditional approaches which yield an average structure, potentially obscuring alternative states that may hold functional significance, and are common in human cells [99]. Deconvolution and annotation of ribonucleic conformational ensembles (DANCE‐MaP) directly addresses this complexity (Table 2). By applying a maximum‐likelihood framework to single‐molecule chemical probing data, DANCE‐MaP simultaneously defines reactivity profiles, thermodynamic populations, and direct base‐pairing and tertiary interactions for up to five co‐existing states from a single experiment conducted in living cells. This is crucial for understanding the implication of RNA functional dynamics on biological processes, as demonstrated by its ability to dissect the ligand‐binding thermodynamics and subtle structural changes of the adenine riboswitch, and to uncover a large‐scale, cell‐state‐responsive structural switch in the human 7SK noncoding RNA, directly linking RNA structural dynamics to the regulation of transcription [100].

A parallel yet distinct challenge in resolving RNA structural landscapes arises from the heterogeneity of RNA isoforms. To date, two long‐read sequencing‐based methods have been developed to analyze the structures of specific isoforms. Nano‐DMS‐MaP and single‐molecule‐based RNA structure sequencing (smStructure‐seq) address this issue by leveraging long‐read Nanopore or single molecule real time (SMRT) sequencing (also known as PacBio sequencing) sequencing respectively, each with specially tailored analytical workflow for chemical probing data analysis (Table 2) [87, 101]. While both methods overcome the limitations of short‐read ensemble averaging by capturing full‐length isoforms and offer different advantages: smStructure‐seq excels in revealing conformational diversity within individual isoforms, whereas Nano‐DMS‐MaP provides a robust and scalable solution for resolving structural differences between highly similar RNA transcript variants in complex biological systems, as demonstrated by resolving spliced and unspliced HIV‐1 transcripts in infected cells.

#### 3.2.3 Direct Detection of Base Pairing

One of the limitations of base‐specific probes is their selectivity toward specific bases or nucleotide states, potentially leading to ambiguity in defining paired RNA regions. This challenge is addressed by direct base‐pairing detection methods such as Pairing Ascertained from Interacting RNA Strands Measured by Mutational Profiling (PAIR‐MaP), which builds on the principle of single‐molecule correlated chemical probing, also employed in the above‐mentioned DANCE‐MaP for ensemble analysis (Table 2) [72]. By utilizing DMS at pH 8.0 to enable probing of all four nucleotides and leveraging mutational profiling for modifications readout, PAIR‐MaP directly identifies base‐pairing interactions through statistically significant correlations in mutation events between complementary sequences on individual RNA molecules. This correlation stems from the destabilization of the helix upon the initial modification of one nucleotide, which increases the probability of modifying its complementary nucleotide. As a result, PAIR‐MaP yields a nucleotide‐resolution, model‐free map of duplex interactions, capable of resolving complex elements like pseudoknots in living cells [37].

#### 3.2.4 Probing RNA Structure Through Solvent Accessibility

While conventional structure probes like DMS and SHAPE primarily report on Watson‐Crick base pairing, Light Activated Structural Examination of RNA by sequencing (LASER‐Seq) and its mutational profiling variant (LASER‐MaP) introduce a distinct approach by measuring nucleobase solvent accessibility at the C8 position of adenines and guanines (Table 2) [102]. Consequently, LASER can detect ligand‐binding sites and conformational changes even within base‐paired regions of highly structured RNAs, such as the ribosome, which presents a challenge for other probes. As such, LASER data complement existing methods and enable the construction of accurate atomistic structural ensembles, thereby enriching the RNA structural toolkit.

### 3.3 Transcriptome‐wide Methods

The escalating demand for large‐scale RNA structure probing has led to an expansion in the methodological scope, transitioning from transcript‐specific analyses to transcriptome‐wide studies utilizing second‐ and third‐generation sequencing technologies (Table 2). These methods have evolved from providing foundational maps to offering mechanistic insights into specific biological processes.

#### 3.3.1 Transcriptome‐wide Structure Mapping

The field of RNA structure probing was transformed by the nearly simultaneous development of DMS‐seq and Structure‐seq in 2014 (Table 2). They not only provided the first transcriptome‐wide views of RNA structure in vivo using DMS in yeast and plants, respectively, but also presented insightful findings of RNA structure in living cells [103, 104]. DMS‐seq demonstrated that mRNAs highly structured in vitro have less secondary structure in living cells, while Structure‐seq highlighted widespread RNA structural conservation across plant transcripts [94, 105]. Structure‐seq2 was introduced not long after as an enhancement of the original Structure‐seq, offering increased sensitivity and read coverage, minimized deleterious by‐products, and reduced ligation bias [106]. The subsequent shift to mutational profiling strategies prompted the development of DMS‐MaPSeq, suitable for transcriptome‐wide and transcript‐specific studies, as witnessed through several applications of the method in heterogeneous telomerase RNA and HIV‐1 RNA structure probing experiments [105, 107, 108].

With the above‐mentioned methods relying on adduct formation, Lead‐seq offered a complementary perspective by utilizing lead(II) acetate‐induced cleavage of single‐stranded regions avoiding base‐specific bias [109]. A different approach was witnessed with selective 2′‐hydroxyl acylation with lithium ion‐based primer extension (SHALiPE), coupled with high‐throughput sequencing (SHALiPE‐seq), which introduced probing of the presence of noncanonical G‐quadruplex (G4) structures using SHAPE reagent NAI (Table 2) [110, 111]. Recently, SHALiPE‐seq has also been reported to study RNA structure with or without protein of interest in conjunction with protein knockdown in cell [112]. Taken together, these methods have laid a solid foundation for transcriptome‐wide canonical and noncanonical RNA structure probing.

#### 3.3.2 RNA Structure Probing Throughout RNA Lifecycle

The evolution of RNA structure probing from static maps to mechanistic dissection is powerfully illustrated by customized methods that isolate structural information at specific stages of the RNA lifecycle, revealing how folding is coupled to gene regulation. To study RNA structure folding in transcription, structural probing of elongating transcripts (SPET‐seq) was designed to capture nascent RNAs, providing a temporal map of co‐transcriptional folding as the RNA is synthesized (Table 2) [113]. For instance, the method demonstrated that bacterial co‐transcriptional folding is not deterministic, but proceeds through kinetic intermediates and competing pathways. Recently, co‐transcriptional structure tracking (CoSTseq), which harnesses MaP rather than RT‐stops approach to reduce sequencing depth requirement, allowed for global base‐pairing status of nascent RNA after transcription in yeast cells [114]. For post‐transcriptional regulation, 5′CAP‐ and 3′poly(A)‐enriched RNA structure sequencing (CAP‐STRUCTURE‐Seq) used specific enrichment to elucidate how structural features at mRNA 5' ends influence processes like miRNA‐mediated cleavage [115]. To unravel the complex folding of tRNAs during translation, tRNA‐Structure‐Seq resolved their modified and highly structured conformations in vivo [116]. It has also been demonstrated that thermodynamically stable tRNAs undergo stress‐induced structural melting, directly linking their conformational integrity to their translational capacity. Overall, these methods reveal that the RNA structure is a dynamic and regulated layer of information that controls gene expression at every stage, from synthesis to decay.

#### 3.3.3 RNA Structure and Cellular Heterogeneity

Common transcriptome‐wide RNA structure probing workflows include bulk population analysis, which often relies on RNA purified from numerous cells, potentially averaging the resulting structural data. To date, the variability in RNA structure between distinct cell types, as well as its influence on cell identity, remain to be studied in detail. However efforts toward this objective have been made in the form of single‐cell structure probing of RNA transcripts (sc‐SPORT), which modifies the DMS‐MaP protocol for single‐cell RNA sequencing, facilitating the concurrent determination of RNA secondary structure and gene expression within individual cells (Table 2) [117]. This advancement addresses the averaging limitations inherent in bulk population analyses and paves the way for exploring the regulatory role of structure in gene expression. Furthermore, achieving subcellular specificity, techniques like demethylase DMS‐MaPseq (DM‐DMS‐MaPseq) and mitochondrial DMS‐MaPseq (mitoDMS‐MaPseq) enable the comparative mapping of RNA structures, such as those of tRNAs and mRNAs, between distinct compartments like the cytosol and mitochondria, revealing how cellular location dictates structural conformation and function [118, 119].

#### 3.3.4 Probing RNA‐RNA Interactions

RNA structurome is defined not only by secondary structure but also by complex intermolecular contacts. Several cross‐linking and sequencing strategies have been developed to map these direct transcriptome‐wide interactions (Tables 1 and 2). Foundational psoralen‐based methods, such as psoralen analysis of RNA interactions and structures (PARIS), sequencing of psoralen crosslinked, ligated, and selected hybrids (SPLASH), and ligation of interacting RNA followed by high‐throughput sequencing (LIGR‐Seq), utilize derivatives like aminomethyltrioxsalen (AMT) to crosslink RNA duplexes in living cells, which are then identified through proximity ligation and high‐throughput sequencing [120, 121, 122]. More recent advancements in RNA‐RNA interaction probing involve the use of amotosalen for PARIS2 [68], a more soluble derivative of AMT, and psoralen‐triethylene glycol azide in the crosslinking of matched RNAs and deep sequencing (COMRADES) [69], facilitating two affinity capture steps and overcoming the low solubility of biotinylated psoralen. As a chemical alternative, SHAPE‐based crosslinking, exemplified by the spatial 2′‐hydroxyl acylation reversible crosslinking (SHARC) approach, uses bifunctional reagents like dipicolinic acid imidazolide (DPI) to covalently link spatially proximal RNAs via their 2'‐OH groups, providing a distinct chemistry for probing transcriptome‐wide tertiary structures and interactions [70]. Lastly, N_3_‐kethoxal was used as part of kethoxal‐assisted RNA–RNA interaction sequencing (KARR‐seq) protocol to probe RNA‐RNA interactions and higher order RNA structures through azide‐dibenzo cyclooctyne (DBCO) click chemistry [46].

#### 3.3.5 Embracing Enrichment Technologies

The field of RNA structure probing continues to be driven by innovations that improve the data coverage and resolution. in vivo click SHAPE (icSHAPE) and its successor icSHAPE‐MaP incorporated click chemistry (with 2‐methylnicotinic acid imidazolide azide (NAI‐N_3_)) for selective biotinylation and enrichment of probed RNAs, improving coverage, particularly at transcript ends (Table 2) [60, 123]. Small amount random RT icSHAPE (smartSHAPE), also based on NAI‐N_3_, addresses the issue of low RNA input, common to low abundance cells, incorporates random RT stops and on‐bead cDNA library construction to reduce sample loss caused by multiple purification steps [124]. Kethoxal sequencing (Keth‐seq), similarly to KARR‐seq mentioned above, utilises N_3_‐kethoxal which not only allows for azide handle modification for enrichment, but adduct of which is also reversible under alkaline or heat conditions, allowing for read‐through controls in RT‐stops detection [44]. Similarly, in vivo click LASER (icLASER) made use of NAz‐N_3_ for enrichment of the modified transcripts, improving specificity and sensitivity of the original method [125].

## 4 Summary and Outlook

Structure probing technologies have revolutionized our understanding of RNA biology by enabling the detailed study of RNA structures within living cells. By leveraging a diverse and expanding toolkit of small‐molecule probes, from base‐specific to nonbase specific and crosslinkers, we can now capture RNA structure, dynamics, and intermolecular interactions directly in living systems. The integration of these structure probes with advanced sequencing technologies for adduct detection has enabled studies at unprecedented scales and resolutions, from single‐cell and subcellular analyses to the dissection of RNA‐RNA interactomes. The synergy between RNA structure probing and sequencing technologies has not only confirmed that RNA function is regulated by its structure but has also revealed previously unknown influences of this structure on RNA's biological functions.

Future chemical and technological advancements are poised to unlock deeper biological insights by enabling precise, spatiotemporally resolved RNA structure probing. It is likely that the field will witness the development of controllable and/or reversible small‐molecule probes, such as those activated by chemical triggers or particular subcellular environments, allowing for time‐ and/or space‐dependent observation of RNA structural transitions.

The future of in vivo RNA structure probing is poised to move from static measurements to dynamic interrogation. This can potentially be achieved by developing a new class of probes, as illustrated by the following two studies on the development of base‐substituting molecules. As highlighted by Shin et al., emissive nucleoside surrogates like ^th^G can be seamlessly incorporated into RNA, functioning as intrinsic, minimally perturbing sensors that enable real‐time monitoring of local conformational changes and biomolecular interactions [126]. The second, more strategic approach is highlighted by Olenginski et al., whose work on the cobalamin riboswitch demonstrates how small molecules can be engineered to bind with high affinity by displacing a nucleobase and engaging a newly revealed cryptic binding site [127]. While this was a targeting effort, the approach can potentially be adopted for RNA structure probing, opening the door to designing a new class of small molecules whose binding is contingent on a specific RNA conformational state. By detecting this state‐dependent binding, perhaps through a fluorescent signal, such molecules can act as exquisitely specific probes for functionally relevant structural transitions. While these two pioneering studies have the potential to become new RNA structure probing small molecules, more studies are needed to tailor these approaches to probing RNA structure in living cells.

Moreover, the integration of artificial intelligence, particularly deep learning, has markedly advanced the prediction of RNA secondary and tertiary structures from sequence alone, as exemplified by methods such as RhoFold and AlphaFold3 [128, 129]. However, these models still face challenges in generalizability, especially for novel or artificial RNA sequences, noncanonical motifs, and complex conformations, partly due to the limited availability of high‐quality experimental RNA structural data [130]. A promising path forward is illustrated by initiatives such as Ribonanza, which combines dual crowdsourcing from the Eterna and Kaggle platforms, to generate chemical mapping data for millions of diverse RNA sequences and uses this large‐scale dataset to train and prospectively evaluate deep learning models. This approach demonstrated how experimental data can enable models to learn realistic representations of RNA structure, including complex features like pseudoknots, even for sequences not seen during training [131]. Large amounts of sequencing data from high‐throughput chemical probing methods provide a powerful resource for understanding RNA structure. By enriching curated databases such as RASP v2.0, which already integrates probing data from diverse sources and includes imputation and prediction tools, the field can acquire the experimental evidence needed to validate and refine AI‐predicted structures [132]. At the same time, pipelines like IPANEMAP Suite hold premise to overcome the complexity of high‐throughput chemical probing data analysis [133]. Ultimately, the continued convergence of chemical biology, sequencing technology, and computational intelligence promises a comprehensive understanding of the RNA structurome, transforming our ability to decipher and manipulate the central role of RNA in life's processes.

## Conflicts of Interest

The authors declare no Conflicts of Interest.

## Data Availability

Data sharing is not applicable to this article as no new data were created or analyzed in this study.
